# Our Vienna Letter

**Published:** 1890-06

**Authors:** Hugh Hagan

**Affiliations:** Vienna


### OUR VIENNA LETTER.

VIENNA, May 1, 1890.

In my last letter I thought there would be no more to say about the late epidemic of Russian Influenza, but there has developed such a number of cerebral and spinal troubles, an increase over any statistical report, without some apparent cause, that the clinicians and pathologists have ascribed these on mental and nervous conditions to the effects of the infective of the epidemic. First and most important, were a series of cases, these are both from reports of private practitioners and the hospital clinics of cerebral abscesses. In all, there were reported about 20 cases, of these 12 occurred in the hospital, and without exception the patients had suffered from one or more attacks of influenza. The ordinary symptoms of cerebral abscess were present, such as rigors, fever, headache, dizziness, epileptic convulsions, etc. Of the 12 cases which I was fortunate enough to be present at the post mortem, 8 had from 1 to 3 abscess cavities in the right frontal lobe, and 3 in the left frontal, only one was situated in the left temporal, andthis case had suffered from inflammation of the middle ear following an attack of influenza. All the eleven cases followed an ordinary history of cerebral abscess. Why the 12th was of particular interest. The patient was found insensible in the street and brought in a comatose condition to the hospital—after being in the hospital 48 hours he regained consciousness and gave the following account, which was corroborated by acquaintances:

He had never experienced any cerebral symptoms up to the morning he left for work. This corresponded to the morning of the noon he was found insensible, when he left his room he felt a slight headache and dizziness, further he knew nothing. From what could be gathered from friends, he had been quite healthy and natural. During the night of the 3d day in the hospital he became suddenly comatose and died before morning. At autopsy was found an abscess in the left temporal, to be almost as large as an ordinary turkey egg. The size of the other abscesses were from a chestnut size to that of a hen egg. The path of infection in first eleven cases was plainly along the filaments of the olfactory nerves through the cribri form plates to the cerebral substance. The twelfth through the mastoid process which was secondary to a middle ear inflammation as sequellae of the influenza. Many cases of myelitis and peripheral neuritis have reported. The myelitis resulting from the infection seems to be of a very tractable type, generally succumbing to the ice bag, ergot and rest. As for the neuretis the majority are very stubborn.

There have been 3 very interesting cases of actinomycosis in the past 6 weeks at the hospital, one being a hostler, the second a butcher and the third a tanner. Direct contagion was traced, and to the astonishment of the investigators, many cases were found in one drove of horses. One of these three cases was especially interesting, as the usual regions affected, the tongue and alveolar processes of the maxilla, were not affected, but the left pleura and ribs there was quite an extensive pyothorax and necrosis of the ribs from the 6th to the 10th, and the abscess pointed just below the nipple.

During this same period two cases of anthrax were examined post mortem. The winter season is now at an end, so to occupy my time as best I might, I secured a letter of introduction from Prof. Benedict to Prof. Winternitz, who is looked upon as the father of hydrotherapy, or in other words, is the famous balneologist of the Polyclinic. He very kindly invited me out to his "Kalt Wasser Amstalt," or in real plain American, his Cold Water establishment.

This offered an opportunity of seeing the most extensive establishment of its kind in Austria, so I availed myself of the opportunity.

The resort (for such is the institution) is situated just 3-4 of an hour from Vienna, in a little village called "*Kaltlentgeben,*" nestled most romantically in a lovely valley in the heart of the mountains, with plenty of clear, cold lime water, and fresh mountain air. Here the Professor has his baths, both hot and cold, steam, hot air, douche, needle, and in short every imaginable mode of applying water to the human body. He is careful never to give the patient too great shock and regulates the temperature to his patient. The lowest temperature that he ever subjects his patients to, is about 8 or 9 centigrade or about 46 F. He is very much opposed to our custom or rather habit of taking ice cold baths in the winter, like many of our citizens do, and who delude themselves by so doing. The "Kalt Wasser Cur," (Cold Water Cure,) consists of nothing but well regulated bathing, good plain diet, exercise, both gymnastic and out door exercise, such as mountain climbing and the like. To carry out this idea of strict and well directed hygiene, he has the Swedish Movement Cure, Massage, a well regulated gymnasium, billiard room, ten-pin alley, tennis, and in short just what is required to develop a healthy organism. Here flocks the anæmic, the chlorotic, the dyspeptic, tabetic, myeletic, melancholic and just such a category of ailing mortals as fill our American capitals to repletion. As you can easily see, the lesson to be taught from a visit to such an institution, is that hygiene in its broadest and most liberal conception, is to be prized above drugs, for I tell you, the leading men in the medical profession of Vienna, lay great stress, not so much on the cold water in itself considered, but, on the life led at such an institution.

Now, for a word about one of our American boys, who has received quite an honorable mention from Professor Schroetter, for being the first one known to him to suggest the easiest mode of securing a good method of examining the larynx for operative purposes.

Dr. L. P. Preston, of Lynchburg, Va., who long wrestled with the bent catheter to elevate the epiglottis, struck upon the happy thought to press upon the middle glosso—epiglottic ligament, when to his great gratification, the epiglottis reared as though spurred, and a full view of the upper air passages was obtained. This method has met with great favor with Professor Schroetter and his assistants.

HUGH HAGAN, M. D.

### A NEW DISINFECTANT FOR SEWAGE.

Mr. Woolheim, a Londoner, is said to have discovered a disinfectant which far surpasses anything now applied for that purpose. This is "amniol," a gas which, when introduced into a sewer, rapidly destroys the microbes of putrefaction and of disease. The odor in the sewer-pipe is almost instantly displaced by that of the gas introduced, and in less than an hour the sewage thus treated is deodorized and sterilized.

Dr. Klein has in part confirmed the claims of the discoverer, in so far that one sample of the sewage examined by him was found to be absolutely sterile after having been treated by the amniol method.

It is to be hoped that further experiments will soon be made with this agent, which will enlighten us as to how long the putrefactive processes can be delayed by it, and the character of microbes it is capable of destroying. If all that is claimed for "amniol" be true, then we will have a new boom in sanitation.—*New Orleans Med. and Surg. Journal.*

CÆSAREAN SECTION.—DR. VINCENT J. HAWKINS, in the *Northwestern Lancet* of February 1st, 1890, reports a successful case of Cæsarean section. The mother was a dwarf, age 17, height 3 feet 5 inches, and measured 30 inches about the hips, and weighed 55 lbs. The baby, a girl, fully developed, weighed at the time of delivery 6 1-2 lbs. The mother and baby, two months after the operation was performed, were doing well, the mother nursing her baby, and were both upon exhibition at the Dime Musuem of St. Paul!—(*Ex.*)

